# Spatiotemporal variation of the indoor mycobiome in daycare centers

**DOI:** 10.1186/s40168-021-01167-x

**Published:** 2021-11-09

**Authors:** Eva Lena F. Estensmo, Luis Morgado, Sundy Maurice, Pedro M. Martin-Sanchez, Ingeborg B. Engh, Johan Mattsson, Håvard Kauserud, Inger Skrede

**Affiliations:** 1 Section for Genetics and Evolutionary Biology (Evogene), Department of Biosciences, University of Oslo, P.O. Box 1066, Blindern, 0316 Oslo, Norway; 2 Naturalis Biodiversity Center, Darwinweg 2, 2333 Leiden, CR Netherlands; 3 Mycoteam AS, P.O. Box 5, Blindern, 0313 Oslo, Norway

**Keywords:** Fungi, Seasonal variation, DNA metabarcoding, Dust mycobiome, Built environment, Microbial ecology, Fungal community

## Abstract

**Background:**

Children spend considerable time in daycare centers in parts of the world and are exposed to the indoor micro- and mycobiomes of these facilities. The level of exposure to microorganisms varies within and between buildings, depending on occupancy, climate, and season. In order to evaluate indoor air quality, and the effect of usage and seasonality, we investigated the spatiotemporal variation in the indoor mycobiomes of two daycare centers. We collected dust samples from different rooms throughout a year and analyzed their mycobiomes using DNA metabarcoding.

**Results:**

The fungal community composition in rooms with limited occupancy (auxiliary rooms) was similar to the outdoor samples, and clearly different from the rooms with higher occupancy (main rooms). The main rooms had higher abundance of *Ascomycota*, while the auxiliary rooms contained comparably more *Basidiomycota*. We observed a strong seasonal pattern in the mycobiome composition, mainly structured by the outdoor climate. Most markedly, basidiomycetes of the orders *Agaricales* and *Polyporales*, mainly reflecting typical outdoor fungi, were more abundant during summer and fall. In contrast, ascomycetes of the orders *Saccharomycetales* and *Capnodiales* were dominant during winter and spring.

**Conclusions:**

Our findings provide clear evidences that the indoor mycobiomes in daycare centers are structured by occupancy as well as outdoor seasonality. We conclude that the temporal variability should be accounted for in indoor mycobiome studies and in the evaluation of indoor air quality of buildings.

Video abstract

**Supplementary Information:**

The online version contains supplementary material available at 10.1186/s40168-021-01167-x.

## Background

Humans spend a significant amount of time indoors, in private homes, but also in workplaces, schools, daycare centers, and hospitals. We share these indoor environments with a variety of microorganisms, including microscopic fungi that may affect our health in different ways. In moist conditions, fungi can propagate and act as sources of indoor pollutants leading to poor indoor air quality. This has been associated with adverse health effects, such as allergies, asthma, and other respiratory symptoms [1, 2]. The indoor microorganisms originate from both indoor and outdoor sources and are potentially structured by numerous factors, including building features, building usage, the number and type of occupants, and, not least, our behavior [3, 4]. The bacterial indoor microbiome is known to be highly affected by the occupants and their activities, and often directly related to the human body [5, 6]. However, indoor fungi, which can be referred to as the indoor mycobiome, are known to be highly influenced by the outdoor air and climate [5, 7, 8]. Previous studies at a large geographical scale in the USA and Norway have demonstrated that the composition of the indoor mycobiomes significantly correlates with variables of the outdoor environment (i.e., climate, soil, and vegetation) [9, 10]. The most important indoor sources of fungi include occupants, pets, food, waste, plants, plumbing systems, mold damages, heating, ventilation, and air conditioning [11]. Different rooms in buildings may have different mycobiome compositions due to different occupancy and exposure to outdoor air [12, 13]. For example, central rooms with higher activity, like the kitchen and living room, promote dust resuspension in the air that facilitates dispersal of fungi from occupants, their activities, and outdoor sources. Similarly, floor dust of high activity rooms contains higher levels of skin-associated yeasts of the genera *Rhodotorula*, *Candida*, *Cryptococcus*, *Malassezia*, and *Trichosporon* [14].

The indoor mycobiomes may differ not only in space but also in time. Previous culture-based studies have been reviewed by Nevalainen et al. [15], where they found a general pattern of seasonal variation with lower concentrations of airborne fungi in winter than in summer. This review included studies from different climatic regions in countries like Australia [16], Denmark [17], and Taiwan [18]. DNA-based studies have also reported a clear seasonal variation of fungal richness, diversity, and community composition in indoor environments, in both dust and air samples [7, 19]. By analyzing dust samples from a university housing facility in California, Adams et al. [7] reported higher fungal richness in winter than in summer. Likewise, Weikl et al. [19] showed a drop of the fungal diversity in summer, based on floor dust samples from 286 houses in Munich. This latter observation was explained by the high prevalence of a few dominant taxa during summer [19]. Hence, the richness response to season varies in these previous studies, which could be a result of the different climates of the sampling localities of the studies. We expect higher indoor fungal richness in the main outdoor fungal growth season.

In boreal and temperate climatic regions, the fungal spore diversity and composition in outdoor air are expected to vary significantly more throughout the year because of distinct seasons. For example, Karlsson et al. [20] reported the lowest richness of fungi and bacteria for air samples collected during winter in two climatic zones from Sweden. It can be expected that this variation influences the indoor mycobiome, due to an influx of spores into buildings. Many fungi, especially basidiomycetes, produces fruit bodies during the fall leading to a relatively higher spore abundance during this period [21]. Plant pathogens, dominated by ascomycetes, may have a wider temporal distribution since many spread asexual spores during the entire plant growth season [22]. Indoor fungi originating from indoor sources, here growing on available organic materials, can be expected to have a year-round growth and sporulation connected to human activity.

A particularly interesting environment to study the spatiotemporal variation of the mycobiome is daycare centers, where children, at least in parts of the world, spend a considerable amount of time. For example, in Norway, 92.2% of children between 1 and 5 years old are in daycares. This particular built environment is characterized by a high occupancy with high levels of activity, and higher fungal concentrations have been detected here compared to private homes [23]. Exploring the indoor mycobiome and revealing the factors driving this spatiotemporal variation are important not only to understand the ecological context of indoor fungi but also to recognize the effect that some fungal species may have on children’s health. To what degree the mycobiome associated with daycares affects the children’s health is still unknown.

The overarching aim of this study is to reveal the indoor mycobiomes’ spatiotemporal dynamics in daycare centers in order to improve evaluations of air quality in indoor air. We expect rooms with different occupancy to differ in mycobiome composition (hypothesis 1; H1), with frequently accessed rooms being dominated by indoor fungi derived from the occupants and their activities. Given that part of the indoor mycobiome originates from outdoor sources, we hypothesize that indoor mycobiomes fluctuate with seasons in temperate and boreal regions (H2). In seasons with optimal fungal growth conditions outdoors, as in summer and fall, we expect that a higher proportion of the indoor mycobiome is derived from outdoor sources, with *Basidiomycota* dominating during the fall season (H3). In contrast, we expect that a higher proportion of the mycobiome has an indoor origin with an increased amount of time spent inside during winter and spring (H4). To test these hypotheses, we collected indoor dust and outdoor air samples from two daycare centers in Oslo, Norway, during a year. We collected dust swab samples every second week from different rooms and stores in the daycare centers, as well as outdoor air samples every week. Fungi present in the samples were surveyed through DNA metabarcoding analyses of the rDNA ITS2 region.

## Results

### Mycobiome composition

**Fig. 1:**
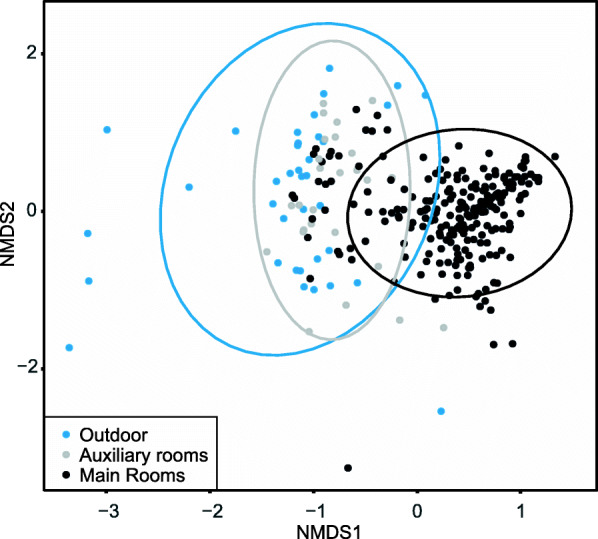
Fungal community composition (Nonmetric multidimensional scaling, NMDS, ordination plot) of outdoors air samples and indoor dust samples from different room types (main and auxiliary) of two daycare centers in Oslo, Norway sampled throughout a full year. Each point represents one sample, and the color separates the samples from the outdoor and the auxiliary and main rooms

**Fig. 2:**
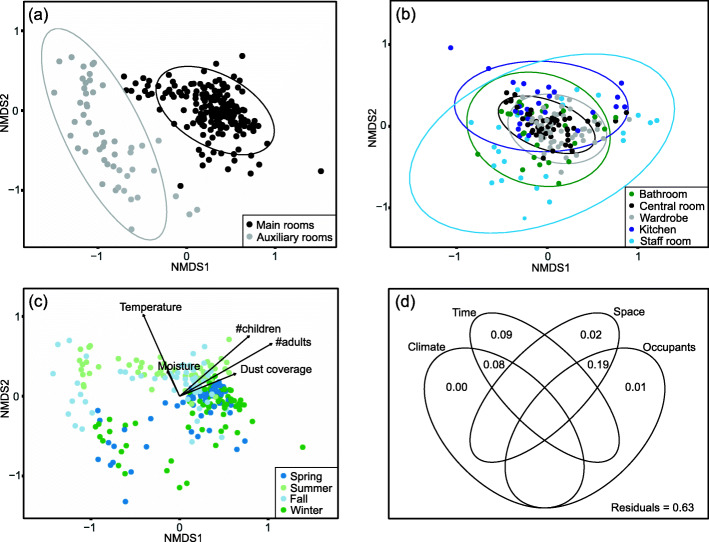
Fungal community composition in indoor dust samples from two daycare centers in Oslo, Norway throughout a full year. **a** NMDS ordination plot of indoor dust samples displaying their compositional variation in the mycobiome. The color differentiates the main rooms from the auxiliary rooms. **b** NMDS ordination plot of main rooms, with colors differentiating between the rooms. **c** NMDS ordination plot of the indoor samples differentiated by season, including numerical variables with significant association (*p* < 0.05). **d** Variation partitioning analysis (VPA) for the indoor dataset (including auxiliary and main rooms), summarizing the effects of four groups of variables: climate = temperature (PCA1) and moisture (PCA2), time = month and season, space = daycare and room, occupants = number of adults, age of children and number of children

The dust mycobiomes from the basement and loft (hereafter called the auxiliary rooms) seemed more similar to the mycobiome obtained from the outside environment than other indoor rooms (hereafter called the main rooms): This could be observed in multivariate analyses (Fig. 1) and from adonis tests (5.6% of the variation was explained comparing outdoor vs auxiliary rooms, and 7.9% of the variation was explained comparing outdoor to main rooms; both *p*-value = 0.001, Supplementary table 1). There was a distinct difference in mycobiome composition between samples from the auxiliary rooms and the main rooms (13.9% of the variation was explained when comparing main rooms vs auxiliary rooms, *p*-value = 0.001, Supplementary table 1; Fig. 2a), the latter used more frequently by the staff and children. Which daycare the samples were collected from explained little of the variation (only 3.3% of the variation was explained when comparing the main rooms of the two daycare centers; Table 1, *p*-value = 0.001). The compositional variation (beta-diversity) across samples was more dispersed in the NMDS plot in some rooms, like the kitchen and staff room (Fig. 2b).

**Table 1:** Adonis test results showing the influence of the variables on the composition of the dust mycobiome from the complete indoor dataset (auxiliary and main rooms), and from the main rooms of two daycare centers sampled in Oslo, Norway throughout a full year. Temperature and moisture are outdoor climatic variables

Variable	Variable information	Auxiliary and main rooms		Main rooms	
		*R* squared	*p*-value	*R* squared	*p*-value
Room	Categorical (9 rooms: 7 main, 2 auxiliary)	0.218	0.001	0.092	0.001
Month	Categorical (12 months)	0.196	0.001	0.233	0.001
Children age	Categorical (1–6 years)	0.133	0.001	0.056	0.001
Season	Categorical (4 seasons)	0.115	0.001	0.124	0.001
Nr of adults	Categorical (4–10 adults)	0.114	0.001	0.032	0.001
Nr of children	Categorical (10–36 children)	0.097	0.001	0.027	0.001
Temperature	Continuous	0.071	0.001	0.082	0.001
Daycare	Categorical (2 daycares)	0.07	0.001	0.033	0.001
Season	Categorical (winter/spring vs summer/fall)	0.062	0.001	0.070	0.001
Dust coverage	Categorical (low, medium, high)	0.039	0.001	0.008	0.029
Moisture	Continuous	0.02	0.001	0.022	0.001

We observed temporal variation in the indoor mycobiome composition (month and season explained 19.6% and 11.5% of the variation in all indoor samples, respectively, *p*-value = 0.001; Table 1; Fig. 2c). Although there was some overlap, the winter and spring samples were more similar in fungal community composition, as were the samples from summer and fall, explaining 6.2% of the variation (*p*-value = 0.001, Table 1). The temporal trend in mycobiome composition correlated with the yearly variation in temperature and moisture, as could be seen from the vectors fitted in Fig. 2c (and further supported by adonis test, *p*-value = 0.001, Table 1).

A variation partitioning analysis of the indoor dust mycobiome (Fig. 2d) revealed that 37% of the compositional variation could be ascribed to assessed factors, including outdoor climate, time (i.e., the biweekly sampling point), space, and occupant characteristics. Most of the explained variation was accounted for by the combined effects of occupants and room type (19%). These two factors are correlated, as the activity of both staff and children are considerably lower in the auxiliary rooms than in the main rooms. Nine percent of the variation was accounted for by time alone, likely reflecting other unmeasured environmental factors changing with time, while 8% was accounted for by the combined effect of time and climate, which again are tightly coupled.

### Taxonomic variation

**Fig. 3:**
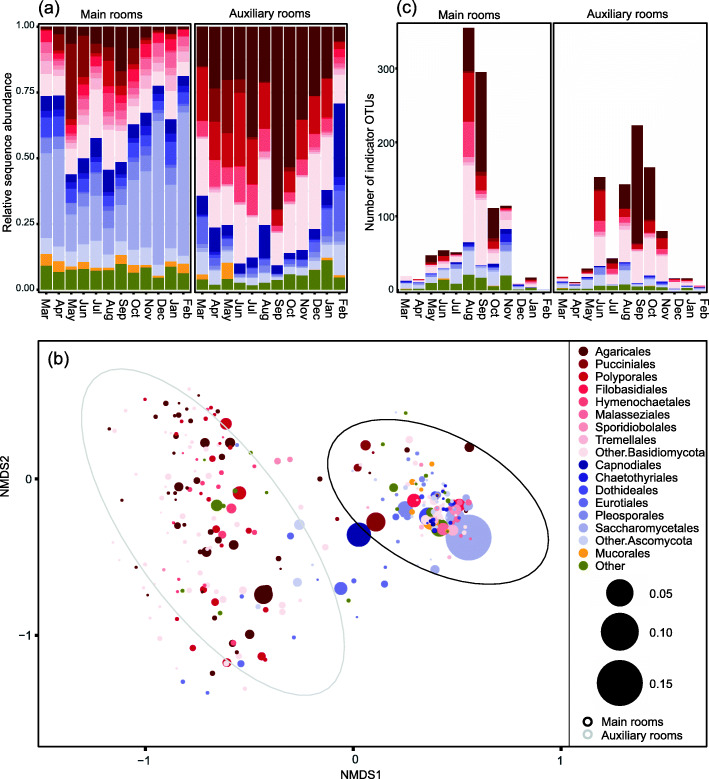
Temporal taxonomic variation in indoor dust samples from two daycare centers in Oslo, Norway sampled throughout a full year. **a** Relative abundance of the main fungal orders. **b** NMDS ordination plot of the 300 most abundant fungal OTUs. Point size indicates relative abundance and colors indicate their taxonomical order. Colors in red = orders belonging to Basidiomycota, blue = orders belonging to Ascomycota, yellow = Mucorales, and green = OTUs belonging to other orders. The ellipses represent the main rooms and the auxiliary rooms, as shown in Fig. 2a. **c** Number of indicator OTUs detected in the indicator species analyses for each month, as well as their taxonomic affiliation at the order level (only OTUs present in at least 3 samples per month were included). Seasons: winter from December to February, spring from March to May, summer from June to August, and fall from September to November

Overall, *Ascomycota* was more prominent in the main room while *Basidiomycota* was far more abundant in samples from the auxiliary rooms (Fig. 3a). The composition of reads from both these two phyla was significantly different in the main vs auxiliary rooms (as observed in ANCOM analyses; Supplementary table 2). This was also the case for *Mortierellomycota*. Ascomycete yeasts affiliated to *Saccharomycetales* were more abundant in the main rooms, while basidiomycetes from the orders *Agaricales* and *Polyporales* were dominating the samples from auxiliary rooms (as seen from the most abundant OTUs; Fig. 3b). We observed a clear temporal trend in the composition of fungal taxonomic groups (displayed at order level in Fig. 3a, and tested with ANCOM across months in Supplementary table 3). Most markedly, the proportion of basidiomycete sequences from the orders *Agaricales*, *Polyporales* and *Hymenochaetales*, mainly reflecting outdoor fungi, were higher during the growth season (May-November) than in winter (Fig. 3a). This pattern was significant across months for the main rooms (Supplementary table 3), but a similar trend was observed in auxiliary rooms. Only *Pucciniales* had a significant temporal trend in the auxiliary rooms (Supplementary table 3). The *Ascomycota* was proportionally more abundant in colder periods, with a significant monthly trend in the main rooms (Fig. 3a; Supplementary table 3). The order *Eurotiales*, including fungal genera with allergenic potentials, such as *Penicillium* and *Aspergillus*, was relatively more prevalent in the main rooms in the colder season (Fig. 3a, Supplementary table 3). Similar trends were observed in the OTU ordination plot (Fig. 3b), where the dominant *Ascomycota* OTUs are associated with the main rooms, while the *Basidiomycota* OTUs with the auxiliary rooms. Further, the main rooms are dominated by OTUs of *Saccharomycetales*, *Mucorales*, *Malasseziales*, and *Filobasidiales*, where *Saccharomycetales* and *Malasseziales* also have a significant monthly seasonality (as seen from the ANCOM analyses, Supplementary table 3).

Indicator species analyses, assessing which fungal OTUs followed a significant temporal trend on a monthly basis, revealed that numerous OTUs in the already mentioned orders of *Agaricales*, *Polyporales* and *Hymenochaetales* increased considerably during their expected fruiting season, independently of space (i.e., room) (Fig. 3c). The significance of each indicator OTU can be found in Supplementary table 4 (auxiliary rooms) and 5 (main rooms).

### Richness and evenness trends

**Fig. 4:**
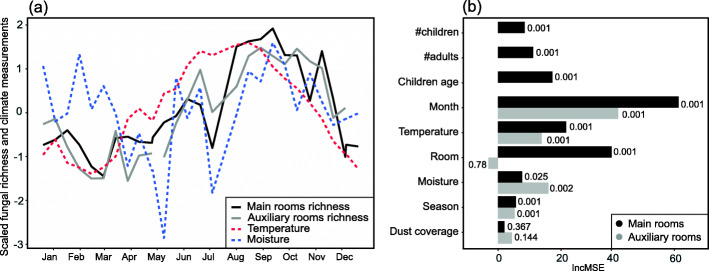
**a** Richness time series for the main room and auxiliary rooms of two daycare centers in Oslo, Norway sampled throughout a full year. The gap in auxiliary room richness in May is due to samples being excluded from the analysis because of the low number of reads. **b** Random forest model showing the importance (percentage of increase in mean squared errors—IncMSE) of each variable for richness of the two indoor datasets, auxiliary and main rooms. Numbers on the bars indicate statistical significance, which was obtained through bootstrapping

The main and auxiliary rooms had a comparable fungal richness that largely followed a similar temporal trend, with higher richness in the summer and fall (June-November) (Fig. 4a). The richness followed the variation in annual temperature. In winter, the richness deviated more from the moisture gradients. The evenness followed a similar trend as the richness (Fig. S3).

A random forest model, which was used to assess the contribution of each factor in the observed richness patterns (Fig. 4b), revealed that month and season (both enclosing various environmental factors), as well as, temperature and moisture, accounted for much of the variation in richness in both the main and the auxiliary rooms datasets. In addition, the factor room was highly important in the main room dataset, where the presence of children and adults also contributes to the richness.

## Discussion

In this study, we observed that the indoor mycobiomes of two daycare centers were strongly structured by room type and occupancy (hypothesis H1) and, further, that the mycobiomes changed systematically throughout the seasons (hypothesis H2). No marked difference in mycobiome composition was observed between the two studied daycare centers, indicating a common pattern of indoor mycobiomes in daycare centers from the same local geographic region.

### Spatial distribution

We observed a clear separation in mycobiome composition of the main rooms and auxiliary rooms, which likely can be explained by the number of people accessing and using the rooms. These results support our hypothesis (H1) and further suggest that occupancy is an important factor shaping the indoor mycobiome, in addition to the outdoor air. The outdoor samples (air sampling) were collected as point samples in one day, while the auxiliary room samples represent a collection of dust accumulated within two weeks. These different sampling methods may influence the recovered mycobiomes. Nevertheless, the mycobiome composition of the auxiliary rooms and the outdoor mycobiome were highly similar, which supports that occupancy strongly affects the indoor mycobiome. Previous studies of indoor environments suggest that the indoor mycobiomes are highly affected by outdoor air [7, 9, 10, 17, 24]. Most of these studies have not accounted for indoor environments with different levels of activity. However, in a recent study in private homes in Norway, we demonstrated that the number of inhabitants affected the indoor mycobiome composition [10].

The highest fungal richness was found in the main rooms. This may be explained that the indoor air of the main rooms includes outdoor taxa, in addition to the more specific indoor fungi derived from the occupants and their activities. Higher fungal richness in indoor environments than in outdoor air has also been found in private houses and schools [5, 10, 14]. It should be noted, though, that richness analyses based on DNA-metabarcoding are vulnerable to various biases. For example, if some dominant species are present, they may mask the remaining richness during the PCR process, since their DNA templates may outcompete the rarer species during PCR amplification. However, the evenness follows largely the same trend for both types of rooms and is therefore probably not causing significant biases for the richness analyses.

OTUs of the phylum *Basidiomycota* were overrepresented in the auxiliary rooms, whereas there were relatively more OTUs of *Ascomycota* in the main rooms. Likewise, previous studies have demonstrated a predominance of *Ascomycota* in indoor samples, while *Basidiomycota* prevails in outdoor samples [25]. As the auxiliary rooms were more similar to the outdoor air, we expected basidiomycetes to be more prevalent in these rooms, especially the mushroom-forming *Agaricales* and *Polyporales*. In the main rooms, the high abundance of ascomycetes can be explained by their high tolerance towards environmental stressors, such as low water availability, a typical condition in indoor environments. The orders *Saccharomycetales* and *Capnodiales* were the most abundant ascomycetes. *Saccharomycetales* are yeasts including the well-known genera *Saccharomyces*, associated with foods, and the potential human pathogen *Candida*. *Capnodiales*, with the widespread genus *Cladosporium*, includes both plant and human pathogens [26]. In addition, the basidiomycete orders *Malasseziales* and *Filobasidiales*, together with the order *Mucorales* were abundant in the main rooms. These orders include yeasts and molds and were also more abundant in indoor mycobiomes than outdoor air in our previous study of private homes [10].

### Seasonality

We observed a clear seasonal pattern in the indoor mycobiomes, supporting our hypothesis H2. Collection month was best able to explain the variation in fungal richness in all rooms. This seasonal pattern is further supported by the evenness and richness analyses of time series, which follows the shift of temperature and moisture throughout the year. Our observed patterns mirror those found in seasonal studies on outdoor mycobiomes. For example, in northern Sweden, the outdoor fungal communities shifted throughout the season [20]. Since the outdoor fungal community has a strong impact on indoor mycobiomes, it is expected that seasonal changes in the outdoor environment also affect which fungi that occurs indoors. During the spring, summer, and fall, with temperatures above zero, fungal activity and sporulation are clearly linked to the level of precipitation (i.e., rainfall). However, during winter, the precipitation manifests largely as snow, which has less effect on the fungal communities at sub-zero temperatures. It has been suggested that during the winter, when the ground is frozen and covered by snow, the impact of the outdoor fungal community on the indoor mycobiome is limited [27]. This can explain the drop of richness during the winter observed in our study.

Although our results demonstrate that dust sampling can be used to reveal the seasonal variation in the indoor mycobiome, there are methodological constraints that should be taken into consideration when analyzing samples with a relatively small amount of DNA. All samples were treated equally in the laboratory; nevertheless, some steps could represent sources of heterogeneity in the dataset, e.g., variability in DNA extraction efficiency among organisms, primer bias to a different fungal taxonomic group, PCR bias, and sequencing errors, which might affect the fungal community. Some of the rooms with limited occupancy, such as the auxiliary rooms and the staff room, had a considerably lower amount of dust and potentially lower amount of DNA. Likewise, the outdoor air samples collected during winter contained a lower amount of DNA, most likely due to a considerably lower number of fungal spores in the air compared to other seasons.

We observed higher abundances of basidiomycetes during summer and fall in all rooms, with a predominance of *Agaricomycetes*, confirming our hypothesis H3. *Agaricomycetes* cover the mushroom-forming species that typically disperse spores during the summer and fall in high-latitude ecosystems. In addition, more indicator species, showing a distinct temporal pattern, were found in these two seasons, in particular from *Agaricales* and *Polyporales*. Thus, high outdoor spore production of basidiomycetes during the summer and fall affects the indoor mycobiome. A high outdoor aerial abundance of basidiomycetes during summer and fall was also observed in northern Sweden [20]. However, our findings are rather opposite to what has been found in seasonal studies in Munich (Germany) and California [7, 19]. Weikl and colleagues explained their observed decline in diversity during the summer in houses in Munich with a few highly abundant OTUs, and not necessarily of lower diversity [19]. Further, in California, the summers are warm and dry, and the mushroom-forming species of the *Agaricomycetes* often fruit during late fall and winter. Thus, all studies may show the same pattern of higher richness of indoor mycobiome during the outdoor sporulation period of basidiomycetes.

The ascomycetes, especially prevalent in indoor conditions, were proportionally more abundant indoor during winter and spring compared to summer and fall. This confirms hypothesis H4. At sub-zero temperatures during winter and spring, fungal growth and sporulation outdoor are reduced in the study area (Oslo, Norway), which will limit the input of basidiomycetes to the indoor environment. Instead, ascomycetes of indoor origin will be more prevalent during this time period. Similar findings were reported in a seasonal study of indoor mycobiomes of four office complexes, where ascomycete molds and basidiomycete yeasts were more common in the spring and winter [24]. In contrast, in another study monitoring airborne fungi in four daycare centers over 12 months through culturing, viable counts of major indoor fungi were significantly lower in the winter [28]. Overall, they found the ascomycetes *Cladosporium*, *Penicillium*, *Alternaria*, and *Aspergillus* to be the most dominating genera. These genera, considered to be some of the most allergenic fungi normally present indoors and outdoors, have also been reported as abundant in other studies [29, 30]. De Ana et al. investigated the seasonal distribution of these species and found that the highest presence of *Aspergillus*, *Cladosporium*, and *Penicillium* in the indoor environment was registered in the fall, whereas *Alternaria* was more frequent in the summer [30]. In our study, the order Eurotiales, including *Penicillium* and *Aspergillus*, was relatively more prevalent in the main rooms in the colder season. In addition, the genera *Saccharomyces*, *Cladosporium*, and *Didymella,* often encountered in indoor environments in other studies [29], were also especially prevalent in the winter. Numerous indoor ascomycetes are known to cause allergies and disease in humans, and it is a concern if these species have a higher prevalence during the winter when the children spend more time inside. In addition, in a previous study of school environments [14], they showed that occupancy contributed more to the allergenic fungal populations in indoor air than outdoor fungi. Understanding this spatiotemporal variation of the indoor mycobiome is important as the time spent inside during the different seasons varies and will reflect how the children are affected by these fungal species.

## Conclusions

In conclusion, our study demonstrates clear differences in the dust mycobiome composition in daycare centers between rooms with different occupancy. The more human activity, the more the indoor mycobiome differs from the outdoor mycobiome composition. To our knowledge, this is the first study that monitors the same rooms and buildings continuously over a full year using a DNA metabarcoding approach. Thus, our results demonstrate how the mycobiome composition follows a strong seasonal trend, mirroring outdoor weather conditions. Knowledge about the seasonal trends will have important implications for monitoring and evaluation of indoor air quality.

## Methods

### Sampling

Dust samples from the daycare centers were collected with floq swabs (Copan Italia spa, Brescia, Italy) and adhesive tapes (Mycotape 2, Mycoteam AS, Oslo, Norway) from 30 × 40 cm^2^ glass plates located 1–2 m above floor level. The swab collected dust from an area of 30 × 30 cm^2^, whereas the tapes sampled dust from 3.8 × 7.5 cm^2^ from the remaining area to calculate the percentage of dust coverage. These samples were collected once for every sampling date. The plates were placed in different rooms and stores in the daycares. Five rooms were sampled in daycare A, and four rooms in daycare B. The plates were sterilized with 85% ethanol after each harvesting, every second week throughout a year. In addition, outdoor air samples were collected every week throughout a year by processing approximately 1800 L air through a 25-mm cassette with a 0.8-μm pore diameter mixed cellulose ester filter (Zefon international, Ocala, FL, USA) by using a Zefon Diaphragm Sampling Pump (Zefon international, Ocala, FL, USA). The time used to obtain the correct volume air varied between 3 and 6 h. The 294 swab and filter samples were stored at − 80 °C until DNA extraction, whereas the adhesive tapes were directly scanned for dust coverage using Epson Perfection V850 Pro (Seiko Epson Corporation, Nagano, Japan). The percentage of dust coverage was calculated with the Olympus Stream v 1.9 software.

### DNA extraction and fungal metabarcoding

DNA from swabs and filter samples were extracted using the E.Z.N.A Soil DNA kit (Omega Bio-tek, Norcross, GA, USA). Nine DNA extraction controls were included, eight starting from clean swabs, and one starting from a clean filter. The swabs and filters were placed in disruptor tubes using sterilized scissors or forceps, respectively, and 800 μL of SLX-Mlus Buffer was added. The samples were homogenized for 2 × 1 min at 30 Hz using TissueLyser (Qiagen, Hilden, Germany) and stored at − 20 °C until further processing. The samples were thawed at 70 °C, following an incubation of 10 min, and homogenized twice for 1 min at 30 Hz using a TissueLyser. The samples were cooled on ice before 600 μL of chloroform was added. Then, the samples were vortexed and centrifuged at 13,000 rpm for 5 min at RT. The aqueous phase was transferred to a new 1.5-mL tube and an equal volume of XP1 Buffer was added before vortexing. The samples were then added to the HiBind DNA Mini Column and further processed by following the manufacturer’s guidelines. The extracted genomic DNA was eluted in 50 μL of elution buffer.

The ITS2 region was targeted by using the forward primer ITS4 (5′-xCTCCGCTTATTGATATG [31]) and the reverse primer gITS7 (5′-xGTGARTCATCGARTCTTTG [32]). The sample barcodes x ranged from 6 to 9 base pairs. The PCR reaction contained 2 μl of DNA template and 23 μl of master mix; 14.6 μl of Milli-Q water, 2.5 μl of 10× Gold buffer, 0.2 μl of dNTPs (25 nM), 1.5 μl of reverse and forward primers (10 μM), 2.5 μl of MgCl2 (50 mM), 1.0 μl of BSA (20 mg/ml), and 0.2 μl of AmpliTaq Gold polymerase (5 U/μl, Applied Biosystems, Thermo Fisher Scientific). For samples with low DNA concentration (weak gel bands), 5 μl of DNA template, and 20 μl of master mix were used. The DNA was amplified by initial denaturation at 95 °C for 5 min, followed by 32 cycles of denaturation at 95 °C for 30 s, annealing at 55 °C for 30 s, and elongation at 72 °C for 1 min. A final elongation step was included at 72 °C for 10 min. PCR products were normalized by using the SequalPrep Normalization Plate Kit (Invitrogen, Thermo Fisher Scientific, Waltham, MA, USA) and eluted in 20 μL of elution buffer.

The resulting 345 PCR products, including technical replicates, DNA extraction controls (starting from clean swabs and filter), negative PCR controls, and mock community (1 ng/μL equimolar DNA concentration from an artificial mix of *Mycena belliarum*, *Pycnoporellus fulgens, Serpula similis*, and *Pseudoinonotus dryadeus*), were processed in a total of four metabarcoding libraries. The technical replicates included DNA from 12 dust samples and were included in each library. The 96 uniquely barcoded PCR products within each library were pooled, and the pools were concentrated and purified using Agencourt AMPure XP magnetic beads (Beckman Coulter, CA, USA). The quality of the purified pools was controlled using Qubit (Invitrogen, Thermo Fisher Scientific, Waltham, MA, USA). The four libraries were barcoded with Illumina adapters, spiked with PhiX, and sequenced in two Illumina MiSeq (Illumina, San Diego, CA, USA) runs with 2 × 250 bp paired-end reads at Fasteris SA (Plan-les-Ouates, Switzerland). The resulting metabarcoding dataset comprised 41,126,514 sequences.

### Bioinformatics

The raw forward and reverse sequences, were demultiplexed independently on a sample basis using CUTADAPT v 2.7 [33], allowing no mismatches between barcode tags and sequence primer, and sequences shorter than 100 bp were discarded. DADA2 [34] was used to filter low-quality sequences, with a maximum expected error of 2.5, and to correct read errors based on a machine learning model built from the sequence data. We then merged the error-corrected forward and reverse sequences using a minimum overlap of 5 bp. Chimeras were filtered out using the bimera algorithm, with default parameters implemented in DADA2 v.12. The resulting 28,346 amplicon sequence variants were further clustered into operational taxonomic units (OTUs) using VSEARCH [35] at 97% similarity. LULU [36] was used with default settings to correct for potential OTU over-splitting. Taxonomy was assigned using BLAST [37] to the final OTU table using the UNITE database [38]. All the negative PCR controls and most of the negative DNA controls were automatically removed during the bioinformatics because the number of sequences was too low. The OTUs of the remaining controls were inspected to assess any contamination issues. Due to the low number of reads in these samples, the OTUs detected in the controls were not removed from other samples in the dataset. The final dataset (excluding controls and replicates) contained 6800 OTUs accounting for 18,694,392 reads from 292 retained samples. The number of reads per sample varied from 470 (from outdoor air during the winter) to 257,599 with a mean value of 65,365. The number of OTUs per sample varied from 3 to 1259.

### Environmental variables

Climatic variables from the outdoor environment were retrieved from The Norwegian Climate and Service Center (https://klimaservicesenter.no/, accessed March 11th, 2020), recorded by the meteorological station at Blindern, Oslo, Norway. The daycare centers are located within a 500-m radius to the meteorological station. The climatic variables included: mean air temperature, mean dew point temperature, max air temperature, min air temperature, mean cloud area fraction, mean water vapor partial pressure, mean surface air pressure, mean wind speed, max relative humidity, mean relative humidity, min relative humidity, humidity mixing ratio, specific humidity, snow coverage, surface snow thickness, amount of precipitation and duration of sunshine. The variables were downloaded for each week throughout the year, and averages for every 2 weeks prior to sampling were calculated and used for seasonal analyses. These variables were studied with principal component analyses (Fig. S1). The results indicated that the first and second dimensions explained a total of 75.6% of the variance. The first dimension was clearly correlated with variables associated with temperature while the second dimension was associated with variables related to humidity and moisture. The coordinates of dimensions 1 and 2 of the PCA analyses were designated as outdoor temperature and moisture, respectively, and used as a surrogate for all the abovementioned climatic variables in downstream analyses. Season was also included as a variable, with related data averaged accordingly. The following months were grouped in four different seasons: winter from December to February, spring from March to May, summer from June to August, and fall from September to November. In addition, the number of children, age of children, and the number of working adults (staff) having access to each daycare center and room between two sampling dates were recorded and included as variables. Continuous variables were scaled using the *scale* function in R.

### Statistical analyses

All statistical analyses were performed in R version 3.6.2 [39] through RStudio (version 1.3.959) unless stated otherwise. We first confirmed the similarity of the technical replicates by nonmetric multidimensional scaling (NMDS) using the *metaMDS* function from the vegan package version 2.4-2 [40] and visualized by ggplot2 [41] (Fig. S2). Then, the complete dataset was rarefied to 1 649 sequences sample-wise, using the function *rrarefy* (vegan). This led to three samples being discarded for downstream statistical analyses, because of shallow sequencing depth in these samples. We then transformed the abundance of OTU per sample table (OTU table) into Hellinger abundance, using the *decostand* function (vegan). The community structure was analyzed using NMDS as described above. A stable solution, for NMDS, was searched with a maximum number of 200 random starts and iterations with the convergence criteria set to stress and/or scale factor of the gradient below 1 × 10e−7, using a Bray-Curtis dissimilarity distance. The community structure was visualized using ggplot2 [41] with the axes transformed into half-change units.

The results showed a clear distinction between outdoor samples and indoor samples, with the exception of auxiliary rooms (the indoor samples belonging to rooms with a very low frequency of occupancy), which showed very similar patterns to the outdoor samples. Considering that the outdoor samples were collected in a different way and time-frame and that outdoor air seasonality was not the main focus of our hypotheses, we decided to focus on the indoor space. Since the indoor samples showed clear segregation between auxiliary rooms and the main rooms, we decided to analyze the indoor data in two separate sets; auxiliary and main rooms together, and only the main rooms. For both datasets, we rarefied all the samples to the sample with the lowest number reads in the respective dataset, 2657 sequences for the auxiliary and main rooms dataset and 3381 sequences in the main rooms dataset. We used the same procedure described above to analyze and visualize community composition. The function *envfit* (vegan) was used to regress the environmental variables onto the Bray-Curtis dissimilarity matrix. The significance of the regression was assessed using 999 permutations. The variables with significant effect were overlaid as vectors in the ordination (NMDS) graphic with arrows pointing in the increasing direction. In addition, we used the function *adonis2* (vegan) with 999 permutations to perform a permutational multivariate analysis of variance to assess the contribution of each environmental variable in explaining variability in the community structure. The Adonis test was also used on the complete dataset to assess the difference between the outdoor, auxiliary, and main rooms (Supplementary table 1). Additionally, we performed variation partitioning analysis using *varpart* (vegan) to assess the interaction and total variability explained by the following groups of variables: climate (temperature and moisture), time (month and season), space (daycare and room), and occupants (number of adults and children, and age of children).

The taxonomic compositional summary was achieved by summing all the rarefied reads, at the order level, within a sample and averaged across the time period. Richness, Shannon-Weaver and evenness indices were determined using the functions, *specnumber*, *diversity* (vegan), and $\frac{Shannon- Weaver}{\log (richness)}$, respectively. Richness and Shannon-Weaver were strongly correlated; we therefore retained richness as a representative of alpha diversity. To estimate the effects of temperature, moisture, season, month, room, dust coverage, number of children and adults, and children age on richness, we conducted linear models followed by analyses of variance as implemented by *lm* and *anova* functions in R [39]. Random forest models with permutations, as implemented in the R package rfPermute [42], with all predictor variables randomly sampled at each tree node, 500 trees and 999 permutations were applied to determine the significance and importance of each variable. In all models, the squared root of richness was used to normalize the response variable.

Analysis of composition of microbiomes (ANCOM) was performed to assess phylum level read abundance significance between the main room and auxiliary rooms. In addition, the phylum- and order-level read abundance significance was assessed across months for both main rooms and auxiliary rooms, independently (Supplementary table 2-3) [43, 44]. The method was implemented in R following the authors’ guidelines (https://github.com/FrederickHuangLin/ANCOM). In brief, rarefied sequence data was summed per sample and phylum or order level, and significance was assumed with alpha = 0.05 using a false discovery rate correction and a cutoff value of 0.8.

We further identified indicator OTUs on a monthly basis for indicator species analyses using the function *multipatt* in the R package indicspecies [45]. We then retained only OTUs with a *p* < 0.05 and present in at least three samples per month. The results were summarized by the number of OTUs per order per month. The full lists of indicator OTUs of the auxiliary rooms and the main rooms are provided in Supplementary table 4 and 5, respectively.

## Supplementary Information

**Additional file 1.**

## Data Availability

All sequencing data, metadata, and scripts used in the bioinformatic analyses and statistics are deposited in Dryad Digital Repository (http://dx.doi.org/).
