# Supplementary material for: Spatiotemporal variation of the indoor mycobiome in daycare centers
Source: Microbiome. 2021 Nov 9;9:220. doi: 10.1186/s40168-021-01167-x (PMC8576891; doi:10.1186/s40168-021-01167-x)
Supplement: Supplementary file 2 — Additional file 1. [file 40168_2021_1167_MOESM2_ESM.zip › Spatiotemporal variation_Supplementary_Microbiome_rerevised.docx]

**Supplementary material**

**Supplementary tables**

**Supplementary table 1.** Adonis test results showing the influence of the variables on the composition of the dust mycobiome from the complete dataset (outdoor, auxiliary and main rooms) throughout a full year. P-value = 0.001 for all tests.

|  | Out vs. Aux vs. Main | Out vs. Aux | Out vs. Main | Aux vs. Main |
| --- | --- | --- | --- | --- |
| Space | 0.108 | 0.056 | 0.079 | 0.139 |

**Supplementary table 2.** Analysis of composition of microbiomes (ANCOM) on phylum level among main and auxiliary rooms, with alpha = 0.05 using false discovery rate correction and cutoff value of 0.8. True indicates statistical significance and false the opposite.

| Phylum | W Main vs. Auxiliary rooms | Main_vs._Auxiliary_rooms |
| --- | --- | --- |
| *Ascomycota* | 5 | TRUE |
| *Basidiomycota* | 5 | TRUE |
| *Chytridiomycota* | 4 | FALSE |
| *Mortierellomycota* | 5 | TRUE |
| *Mucoromycota* | 3 | FALSE |
| Unknown | 5 | TRUE |

| Taxon | W Main rooms | Main rooms | W Auxiliary rooms | Auxiliary rooms |
| --- | --- | --- | --- | --- |
| *Ascomycota* | 6 | TRUE | 2 | FALSE |
| *Basidiomycota* | 6 | TRUE | 2 | FALSE |
| *Chytridiomycota* | 6 | TRUE | NA | NA |
| *Glomeromycota* | 4 | FALSE | NA | NA |
| *Mortierellomycota* | 4 | FALSE | 2 | FALSE |
| *Mucormycota* | 3 | FALSE | 0 | FALSE |
| *Agaricales* | 17 | TRUE | 12 | FALSE |
| *Capnodiales* | 12 | FALSE | 4 | FALSE |
| *Chaetothyriales* | 16 | TRUE | 0 | FALSE |
| *Dothideales* | 16 | TRUE | 1 | FALSE |
| *Eurotiales* | 15 | TRUE | 10 | FALSE |
| *Filobasidiales* | 6 | FALSE | 0 | FALSE |
| *Hymenochaetales* | 17 | TRUE | 11 | FALSE |
| *Malasseziales* | 14 | TRUE | 2 | FALSE |
| *Mucorales* | 8 | FALSE | 0 | FALSE |
| Other orders | 13 | FALSE | 13 | FALSE |
| Other *Ascomycota* orders | 14 | TRUE | 3 | FALSE |
| Other *Basidiomycota* orders | 15 | TRUE | 8 | FALSE |
| *Pleosporales* | 13 | FALSE | 1 | FALSE |
| *Polyporales* | 17 | TRUE | 6 | FALSE |
| *Pucciniales* | 17 | TRUE | 16 | TRUE |
| *Saccharomycetales* | 17 | TRUE | 0 | FALSE |
| *Sporidiobolales* | 13 | FALSE | 0 | FALSE |
| *Tremellales* | 14 | TRUE | 0 | FALSE |

**Supplementary table 3.** Analysis of composition of microbiomes (ANCOM) on taxon level read abundance across months for main and auxiliary rooms, with alpha = 0.05 using false discovery rate correction and a cutoff value of 0.8. True indicates statistical significance and false the opposite. NA indicates too few reads for the test.

**Supplementary table 4**. The complete list of indicator species OTUs detected for the different months in the auxiliary rooms of a daycare center in Oslo, Norway.

Excel table “Supplementary table 4”.

**Supplementary table 5**. The complete list of indicator species OTUs detected for the different months in the main rooms of two daycare centers in Oslo, Norway.

Excel table “Supplementary table 5”.

**Supplementary figures**

**Fig. S1**. Principal Component Analysis (PCA; Axes 1 and 2) for data from the meteorological numerical variables explored in this study. Data were recorded by the meteorological station at Blindern, located within 1 km^2^ of both daycare centers in Oslo, Norway.


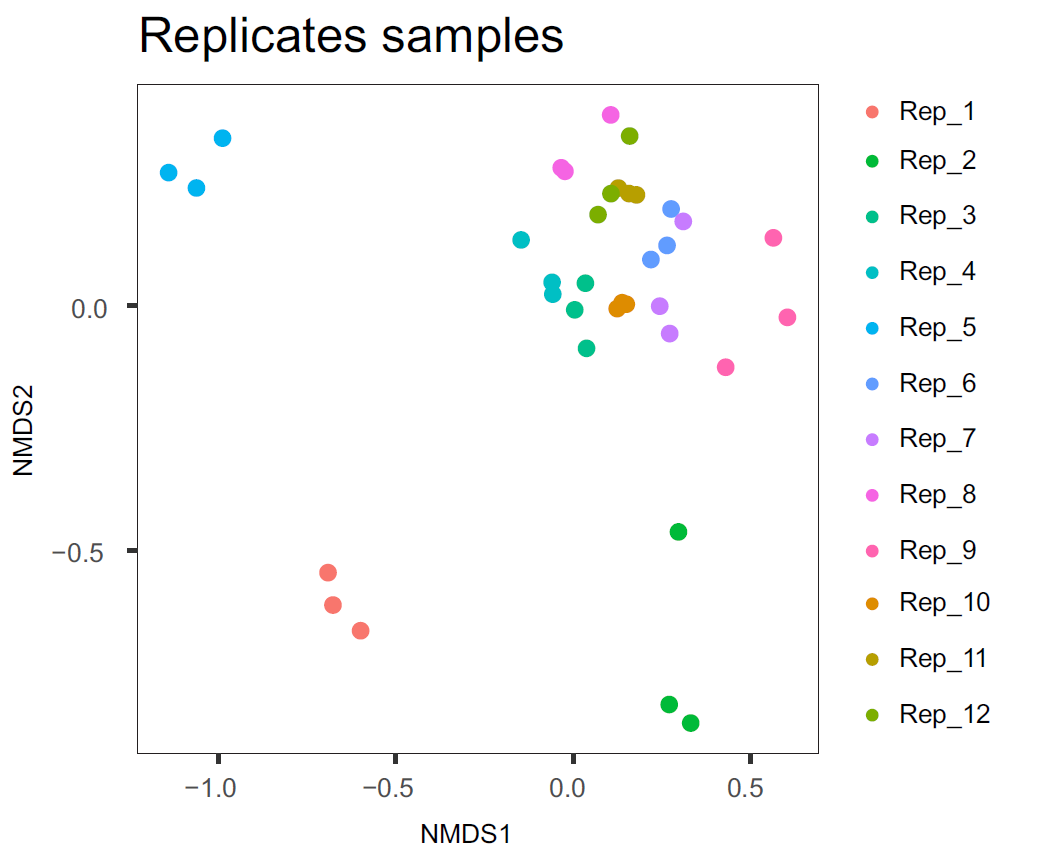


**Fig. S2**. Nonmetric multidimensional scaling (NMDS) ordination plot of technical PCR replicates included in the library preparation and sequencing. Each point represents one sample, and the color indicates the different replicates.


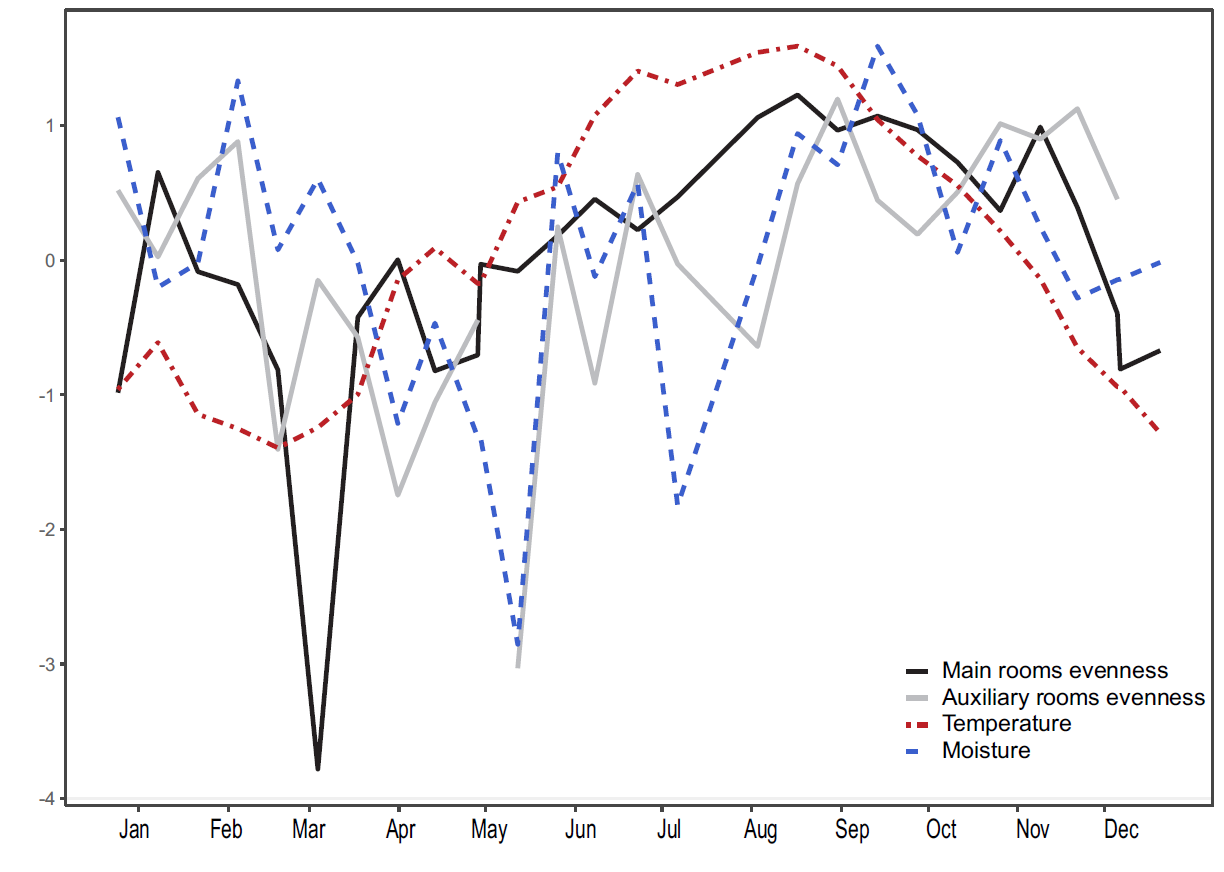


**Fig. S3**. Monthly evenness time-series of the dust samples in the main rooms (black line) and the auxiliary rooms (grey line) sampled in two daycare centers in Oslo, Norway. The dotted lines represent the monthly fluctuation of temperature (red) and moisture (blue). The gap in auxiliary rooms evenness in May is due to samples excluded from the analysis because of low number of reads.
